# The course of skull deformation from birth to 5 years of age: a prospective cohort study

**DOI:** 10.1007/s00431-016-2800-0

**Published:** 2016-11-04

**Authors:** Leo A van Vlimmeren, Raoul HH Engelbert, Maaike Pelsma, Hans MM Groenewoud, Magda M Boere-Boonekamp, Maria WG Nijhuis-van der Sanden

**Affiliations:** 1 Department of Rehabilitation, Paediatric Physical Therapy, Radboud university medical center, P.O. 9101, 6500 HB Nijmegen, The Netherlands; 2 Radboud Institute for Health Sciences, IQ healthcare, Radboud university medical center, P.O. 9101, 6500 HB Nijmegen, The Netherlands; 3 ACHIEVE, Center of Applied Research, Faculty of Health, University of Applied Sciences, Tafelbergweg 51, 1000 CN Amsterdam, The Netherlands; 4 Academic Medical Center, Department of Rehabilitation, University of Amsterdam, Meibergdreef 9, 1105 AZ Amsterdam, The Netherlands; 5 Department for Health Evidence, Radboud university medical center, P.O. 9101, 6500 HB Nijmegen, The Netherlands; 6 Department Health Technology and Services Research, Institute for Governance Studies, University of Twente, P.O. 217, 7500 AE Enschede, The Netherlands

**Keywords:** Deformational brachycephaly, Deformational plagiocephaly, Newborns, Skull deformation

## Abstract

In a continuation of a prospective longitudinal cohort study in a healthy population on the course of skull shape from birth to 24 months, at 5 years of age, 248 children participated in a follow-up assessment using plagiocephalometry (ODDI—oblique diameter difference index, CPI—cranio proportional index). Data from the original study sampled at birth, 7 weeks, 6, 12, and 24 months were used in two linear mixed models. Main findings: (1) if deformational plagiocephaly (ODDI <104%) and/or positional preference at 7 weeks of age are absent, normal skull shape can be predicted at 5 years of age; (2) if positional preference occurs, ODDI is the highest at 7 weeks and decreases to a stable lowest value at 2 and 5 years of age; and (3) regarding brachycephaly, all children showed the highest CPI at 6 months of age with a gradual decrease over time.

*Conclusion*: The course of skull deformation is favourable in most of the children in The Netherlands; at 5 years of age, brachycephaly is within the normal range for all children, whereas the severity of plagiocephaly is within the normal range in 80%, within the mild range in 19%, and within the moderate/severe range in 1%. Medical consumption may be reduced by providing early tailored counselling.

**Table:** 

**What is Known:** • *Skull deformation prevalence increased after recommendations against Sudden Infant Death Syndrome*, *little is known about the longitudinal course.* *• Paediatric physical therapy intervention between 2 and 6 months of age reduces deformational plagiocephaly at 6 and 12 months of age.*
**What is New:** **•** *The course of skull deformation is favourable in most of the children in The Netherlands*; *at 5 years of age*, *deformational brachycephaly is within the normal range for all children*, *whereas the severity of deformational plagiocephaly is within the normal range in 80%*, *within the mild range in 19%*, *and within the moderate to severe range in only 1%.* ***•*** *Paediatric physical therapy intervention does not influence the long-term outcome*; *it only influences the earlier decrease of the severity of deformational plagiocephaly.*

**Electronic supplementary material:**

The online version of this article (doi:10.1007/s00431-016-2800-0) contains supplementary material, which is available to authorized users.

## Introduction

Since epidemiological studies have showed that prone and side sleeping were major risk factors for sudden infant death syndrome (SIDS) [12, 14], supine sleeping has increased, consistent with the recommendations of the American Academy of Paediatrics [2, 3, 13, 15]. Simultaneously, a prevalence increase of skull deformation has also been observed [4, 6, 7, 19, 29]; asymmetrically, this is described as deformational plagiocephaly (DP) and/or symmetrically, which is described as deformational brachycephaly (DB) [4, 19, 32, 34].

The prevalence of DP and DB increases rapidly in young children during the first months of life [19, 21, 34, 39]. DP is attributed to perinatal factors [16, 21, 26, 34, 39] as well as factors in early infancy [6, 17, 19, 39]. Familial and ethnic factors are supposed to be related to skull deformations [25, 31, 34]. Positional preference, when children lie on their back, is the major cause of these skull deformations [4, 7, 19, 34]; children keep their head turned with the same spot on the surface, which slows down growth in that direction and stimulates growth in the other directions [4, 7, 18, 34]. Many clinicians consider skull deformation to be a minor and purely cosmetic condition [11, 20]. Hutchison et al. reported that 4% of skull deformations remained severe at 3 to 4 years of age [20]. In a cross-sectional study, Roby et al. found a prevalence of DP of 1% and DB of 1.1% in 15-year-old teens [33]. Of these children with DP or DB, 38.1% was noted to have abnormal facial characteristics [33].

In children with DP and DB, several conservative interventions are applied: (paediatric) physical therapy [5, 9, 43], helmet therapy [18, 24, 27, 32, 36, 37, 46], manual therapy [8], osteopathy [35], and surgical intervention [22, 30].

The aim of the present study was to investigate the long-term course of skull shape in healthy newborns until the age of 5.5 years, with special interest in the subgroups of children with and without positional preference at 7 weeks, and in the children with positional preference who received paediatric physical therapy intervention (PPT) or not (no PPT).

## Methods

This study provides additive follow-up data of a prospective cohort study with an embedded randomised controlled trial to assess the effect of paediatric physical therapy, with measurements at birth, 7 weeks, 6 and 12 months of age. The additive data of the measurements in children at 2 and 5.5 years of age are presented in this article.

### Participants

**Fig. 1:**
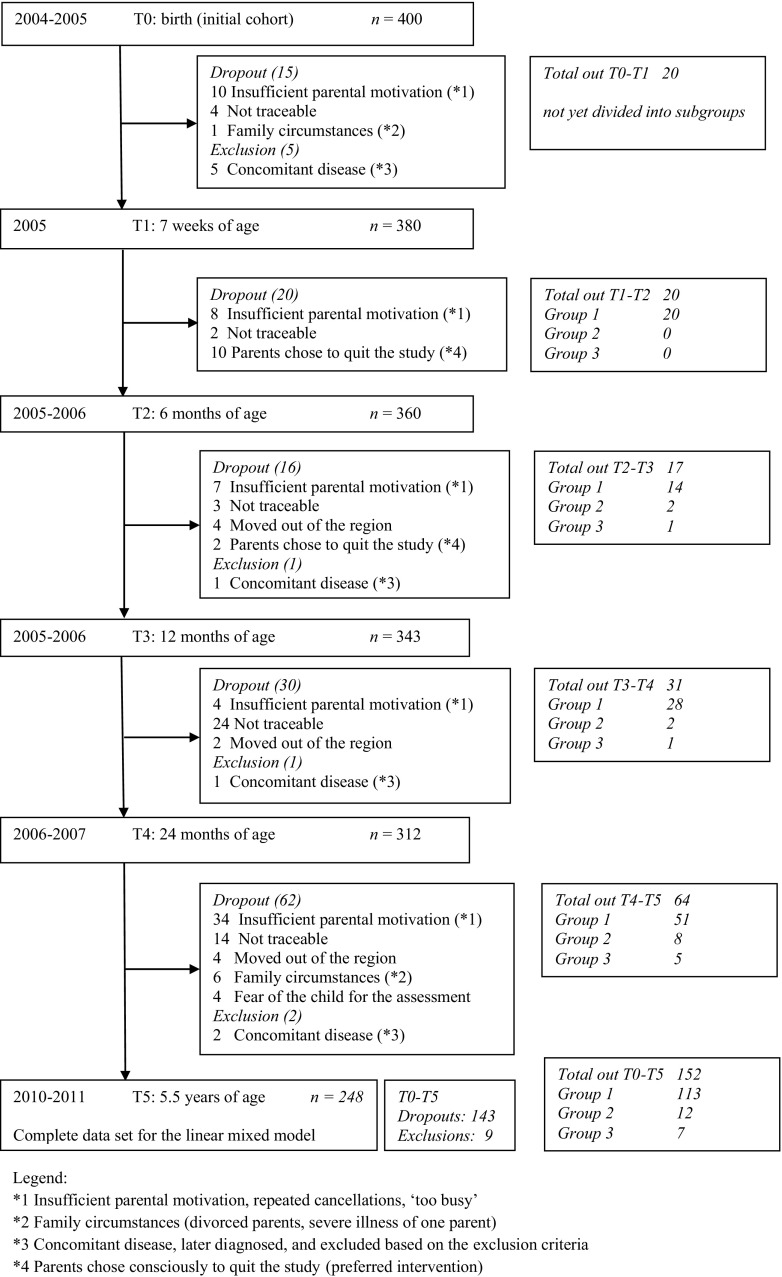
Flowchart of the children assessed six times from birth to 5.5 years of age

The original prospective cohort study started with 380 healthy newborns (≥36-weeks gestation), born between December 2004 and September 2005 at the general district hospital Bernhoven in Veghel, The Netherlands. Children with congenital muscular torticollis (torticollis with a one-sided shortening of the sternocleidomastoid muscle; Kaplan type 2 and 3 [23, 39, 40]), dysmorphism, or syndromes were excluded. A flowchart of included and excluded children over time is presented in Fig. 1. At 7 weeks of age, the embedded randomised controlled trial started and the cohort of children was divided into three groups: (1) children without positional preference (*n* = 315), (2) children with positional preference (*n* = 65) and randomly allocated to PPT (*n* = 33), and (3) children with positional preference and randomly allocated to no PPT (*n* = 32). Results of the RCT until the age of 12 months are presented elsewhere: PPT between 2 and 6 months of age was established to be effective in children with positional preference in reducing DP at 6 and 12 months of age [42, 43]. Therefore, we decided to present the long-term outcome at 2 and 5.5 years for the three above-mentioned subgroups.

### Measures

Participating children were measured at birth (T0), 7 weeks (T1), 6 months (T2), and 12 months (T3). Long-term outcome data were collected at 24 months (T4) and 5.5 years (T5) of age.

#### General characteristics and risk factors

General characteristics including gender, birth rank, parental age, parental educational level, and obstetric data including gestation, pregnancy rank, presentation at delivery, mode of delivery, length of labour, multiple birth, Apgar score, birth weight, and birth head circumference were collected within 48 h of birth.

Gender, being firstborn, nursing, feeding, sleeping, and playing positioning habits (positional preference when sleeping, head to the same side on a chest of drawers, only bottle-feeding, positioning to the same side during bottle-feeding, ‘tummy time’ when awake <3 times per day, and slow achievement of motor milestones with the presence of DP) were considered as potential risk factors, based on a previous study [42].

#### Primary outcome measure

The transversal shape of the skull was measured at all six assessments (T0–T5) by plagiocephalometry (PCM), which is a reliable, valid and responsive instrument [27, 38, 41]. PCM measures the relationship between the transverse shape of the skull and the position of both ears and nose, and thereby the location and amount of flattening of the skull. PCM assesses the severity of DP by the parameter oblique diameter difference index (ODDI: ratio between both oblique diameters of the head) and the severity of DB by the parameter cranio proportional index (CPI: ratio between the width and length of the head). Based on psychometric analysis in a previous study [41] and analogous to other relevant studies [44, 45], we showed that clinically relevant asymmetrical (DP) skull flattening was present in the case of ODDI ≥104%, and symmetrical (DB) skull flattening was present in the case of CPI ≥90%. Furthermore, we defined four categories of skull deformation, whereas ODDI refers to DP, and CPI refers to DB: (1) normal: ODDI <104 and/or CPI <90, (2) mild: ODDI 104–107 and/or CPI 90–94, (3) moderate: ODDI 108–111 and/or CPI 95–99, and (4) severe: ODDI ≥112 and/or CPI ≥100.

PCM was performed by two very experienced examiners who were blinded for the group belonging and who were interchangeable (LV author, FG acknowledgements) [41–43]. The environmental conditions (temperature, light, positioning) during the assessments were the same for all children.

#### Paediatric physical therapy intervention

In 65 children with positional preference, PPT was indicated as described previously [43]. In 33 children, a standardised PPT program was executed due to randomisation of the study between 2 and 6 months of age. The PPT program consisted of exercises to reduce positional preference and to stimulate motor development, by counselling parents on positioning, handling and nursing, supported by a leaflet with basic preventive advice. PPT was stopped when the positional preference no longer occurred during the day and night, when awake and asleep, when the parents were shown to have incorporated all of the advice and exercise in daily handling, and when there were no indications for motor developmental problems (delays or asymmetries). The parents of the control group (no PPT) received only a leaflet with basic preventive advices, without further education to intervene. Both groups received regular advice from well-baby clinics, like every child in The Netherlands [43].

### Statistics

Descriptive statistics were used to analyse baseline characteristics. Means and standard deviations or proportions were calculated for the relevant variables. In the present study, we assessed the association between peri- and postnatal factors, and skull deformation data at 7 weeks of age with the skull deformation at 24 months and 5.5 years of age. The relationship between these factors and deformity was analysed by means of cross-tabulation, as well as linear and logistic regression. In the univariate analyses, putative risk factors with a *P* < 0.15 were selected for inclusion in multivariate models. In the linear regression analysis, the effect of these factors on the dependent factors ODDI (continuous) and CPI (continuous) was assessed at 24 months and 5.5 years of age.

To describe the primary outcome measures ODDI and CPI, two linear mixed models were constructed. One model had ODDI as the dependent variable (related to DP) and the other had CPI as the dependent variable (related to DB). We included time, positional preference at 7 weeks of age and the outcome measures ODDI and CPI at birth as independent variables. The models with the three subgroups, as illustrated in the design, included interactions between positional preference and measurements in time. This time-by-positional preference interaction showed whether there was a difference between the groups over the study period. Time, positional preference at 7 weeks of age, independent ODDI or CPI variables at birth, and the interaction term between positional preference and time were all entered in the models as fixed factors. The ODDI pattern is based on a chosen ODDI at birth of 101. The CPI pattern is based on a chosen CPI at birth of 79.

Residual plots from the mixed models were examined to check model assumptions. Both linear mixed model analyses were performed on the three subgroups.

The mean (95% confidence interval) ODDI or CPI were computed at each time point for each group for a given value of the ODDI and CPI at birth. These parameters also enabled us to estimate the difference between the three positional preference groups at each time point, corrected for the score of the dependent variable at birth. Although not all parameters in both models showed a significant difference from 0, we kept all variables in the model for reasons of consistency.

Analyses were executed as two-tailed with a significance level of 5%. When applicable, 95% confidence intervals were computed. Statistical analyses were performed using SAS software version 9.2 and IBM SPSS Statistics 20.0 software.

## Results

### General characteristics of the study population

We included 380 healthy newborns in the cohort and assessed them shortly after birth. Of these, 248 children (65%) with a mean age of 5.51 years (standard deviation 0.19 years) were analysed at T5: there were 202 children without positional preference, 21 children with positional preference allocated to PPT, and 25 children with positional preference allocated to no PPT. General characteristics of the three included groups at T1 (*n* = 380) and relevant determinants for DP and DB at later age are presented in Table 1.

**Table 1:** General characteristics (*n* = 380) of the three included groups at T1 (7 weeks of age. Data are presented as *n* (%) or mean (standard deviation [SD])

	Group 1 No positional preference (*n* = 315)			Group 2 Positional preference and randomly allocated to PPT (*n* = 33)			Group 3 Positional preference and randomly allocated to no PPT (*n* = 32)		
	*n*	%		*n*	%		*n*	%	
Gender, boy	138	43.8		20	60.6		20	62.5	
First pregnancy	112	35.6		16	48.5		14	43.8	
Delivery									
Vaginal	205	65.1		23	69.7		19	59.4	
Vacuum-assisted	35	11.1		4	12.1		4	12.5	
Caesarean section	75	23.8		6	18.2		9	28.1	
Birth rank									
First born	141	44.8		17	52		16	50.0	
Later born	174	55.2		16	48.5		16	50.0	
Tummy time till 7 weeks of age (T1)									
<5 min per session	206	65.4		26	78.8		25	78.1	
5 to 15 min per session	75	23.8		5	15.2		5	15.6	
>15 min per session	34	10.8		2	6.1		2	6.3	
	*n*	Mean	SD	*n*	Mean	SD	*n*	Mean	SD
Age (years) at birth from									
Mother	315	31.1	4.30	33	30.2	3.35	32	31.4	3.88
Father	311	33.8	4.87	33	33.7	4.97	32	33.6	5.03
Skull circumference at birth (cm)	315	34.7	1.44	33	35.2	1.37	32	34.9	1.17
Birth weight (kg)	315	3.3	0.48	33	3.5	0.44	32	3.5	0.45
Gestation (weeks)	315	39.4	1.48	33	39.7	1.46	32	39.5	1.43
Length of labour, second stage (hours)	242	0.51	0.47	28	0.62	0.59	24	0.65	0.61

*PPT* paediatric physical therapy intervention

The reasons for dropouts and exclusion are illustrated in Fig. 1. Independent *T* tests showed no significant differences in gender and positioning, as well as in ODDI and CPI per subgroup, at each of the earlier time points between participants at T5 and children who dropped out before T5. No other treatments, except PPT in the intervention group, were applied.

### Risk factors identified at 7 weeks of age

There was no association between the potential risk factors nursing, feeding, sleeping, and playing positioning habits at 7 weeks of age and skull deformity at 24 months and 5.5 years of age. At 24 months of age, there was a univariate association between the time spent playing prone (‘tummy time’) measured at 7 weeks of age and the ODDI percentage (*β* = −0.304, *P* = 0.062, 95% CI = −0.624 to 0.015). In the univariate analyses, no putative risk factors with a *P* < 0.15 could be found to be associated with the PCM measurements at 5.5 years of age.

### Primary outcome

**Fig. 2:**
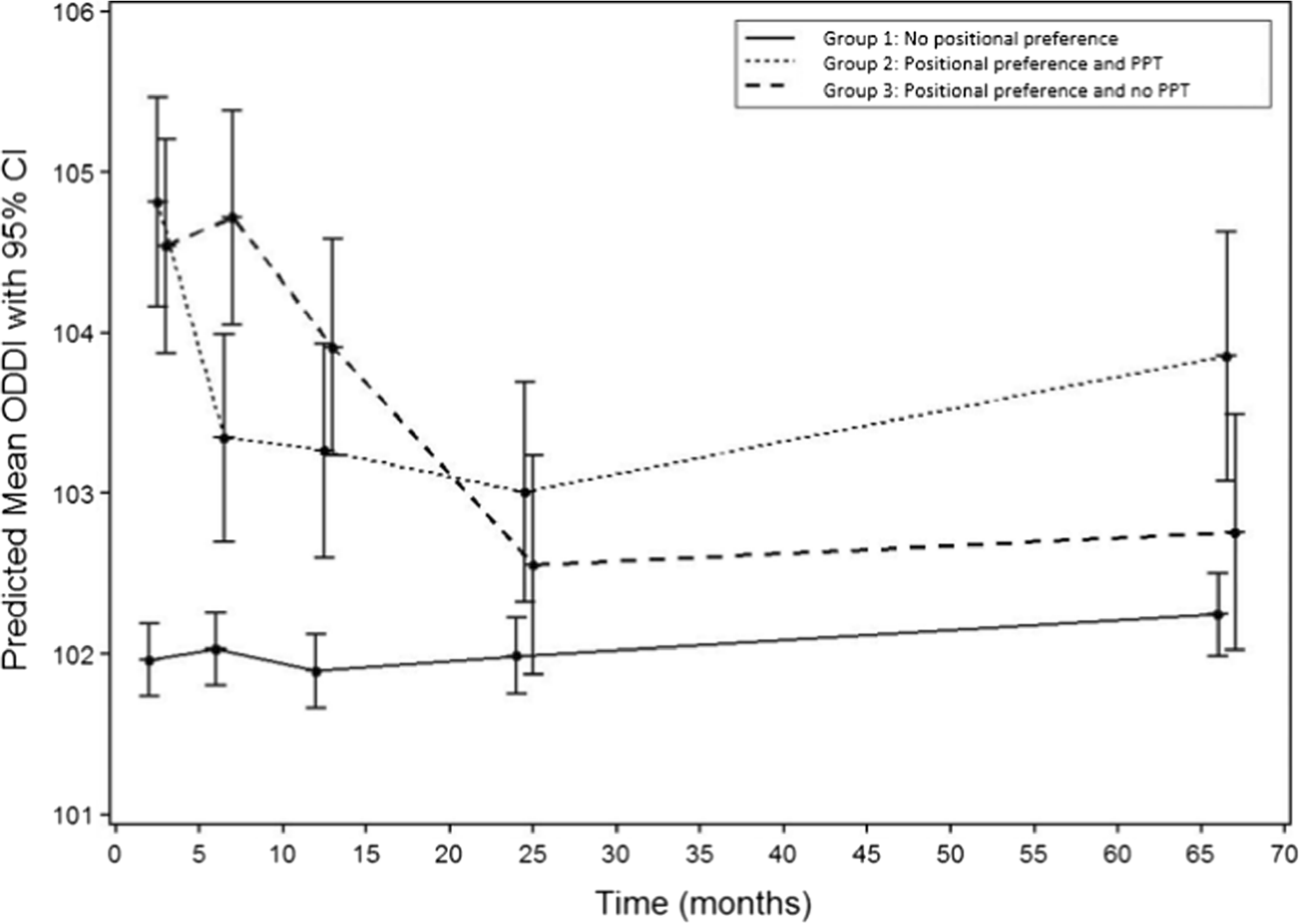
Patterns of the predicted mean ODDI and its 95% confidence interval for the three subgroups

**Fig. 3:**
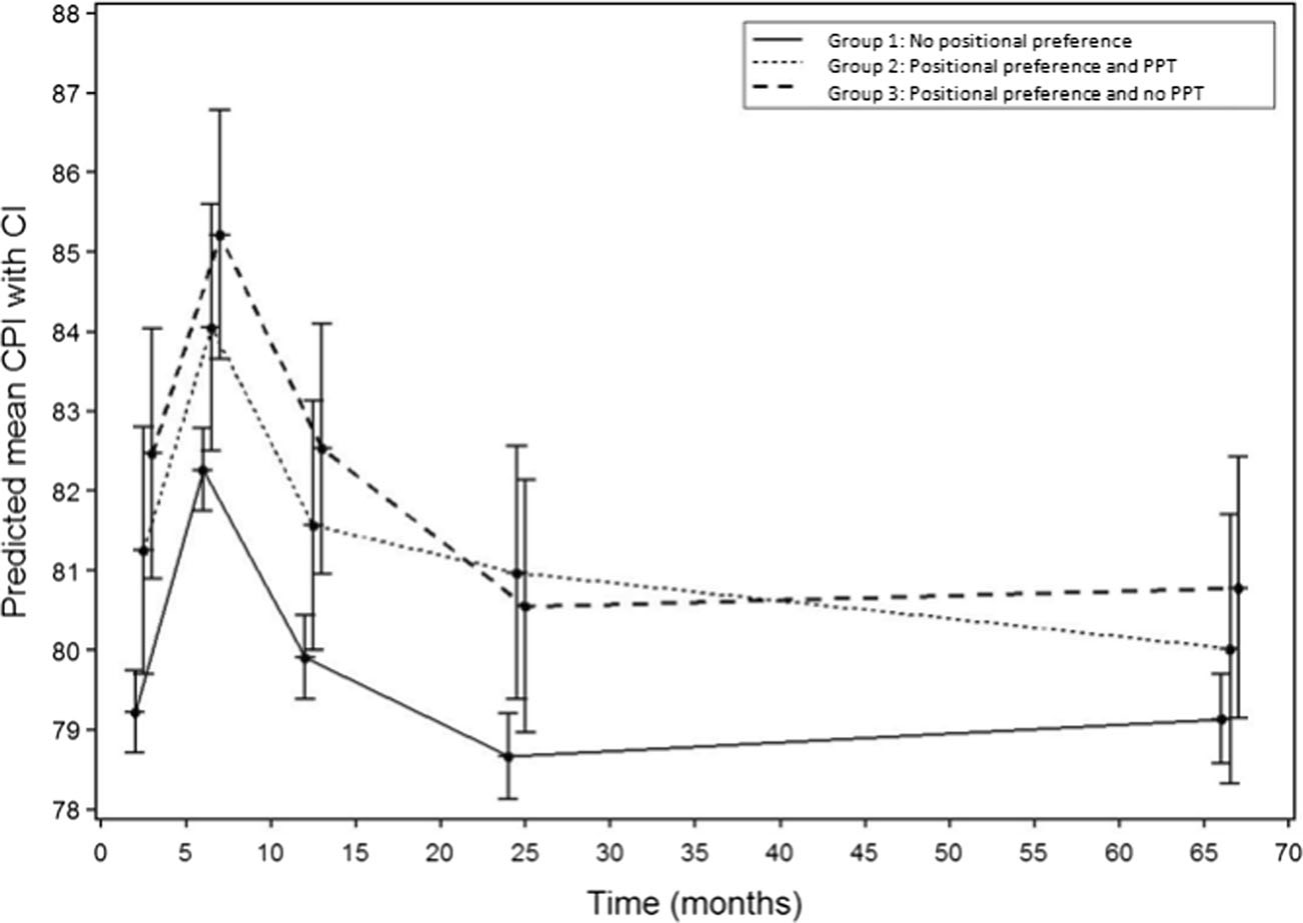
Patterns of the predicted mean CPI and its 95% confidence interval for the three subgroups

The courses of DP and DB over time in the three groups are illustrated in Table 2 and Figs. 2 and 3. Skull deformation regarding DP at T5 in children without positional preference occurred in 17.3% (35 out of 202) at T5. None of the children without positional preference showed DB at T5. In the PPT group, 8 out of 21 (38%) and in the no PPT group, 8 out of 25 (32%) children with positional preference still showed DP at T5. Only two children with positional preference showed DB at T5: mild DB was reported in the no PPT group.

**Table 2:** The course of deformational plagiocephaly (represented by ODDI%) and deformational brachycephaly (represented by CPI%)

	No positional preference							Positional preference and allocated to PPT							Positional preference and allocated to no PPT						
ODDI ➔				Normal	Mild	Moderate	Severe				Normal	Mild	Moderate	Severe				Normal	Mild	Moderate	Severe
Age at assessment	N	Mean	SD	*n* (%)	*n* (%)	*n* (%)	*n* (%)	*N*	Mean	SD	*n* (%)	*n* (%)	*n* (%)	*n* (%)	*N*	Mean	SD	*n* (%)	*n* (%)	*n* (%)	*n* (%)
T0: birth	315	101.6	1.55	298 (94.6)	14 (4.4)	3 (1.0)	0 (0.0)	33	101.5	1.58	30 (90.9)	3 (9.1)	0 (0.0)	0 (0.0)	32	102.1	1.77	29 (90.6)	3 (9.4)	0 (0.0)	0 (0.0)
T1: 7 weeks	315	102.1	1.96	269 (85.4)	42 (13.3)	3 (1.0)	1 (0.3)	33	104.8	2.93	15 (45.5)	14 (42.4)	3 (9.1)	1 (3.0)	32	104.6	2.58	12 (37.5)	18 (56.3)	2 (6.3)	0 (0.0)
T2: 6 months	295	102.0	1.68	266 (90.2)	27 (9.2)	2 (0.7)	0 (0.0)	33	103.6	2.28	23 (69.7)	9 (27.3)	1 (3.0)	0 (0.0)	32	104.8	3.15	14 (43.8)	13 (40.6)	4 (12.5)	1 (3.1)
T3: 12 months	281	101.9	1.71	245 (87.2)	34 (12.0)	2 (0.7)	0 (0.0)	31	103.3	2.99	23 (74.2)	7 (22.6)	0 (0.0)	1 (3.2)	31	103.8	2.40	14 (45.2)	16 (51.6)	1 (3.2)	0 (0.0)
T4: 24 months	253	102.0	1.69	227 (89.7)	23 (9.2)	2 (0.7)	1 (0.4)	29	103.2	2.35	20 (69.0)	8 (27.6)	1 (3.4)	0 (0.0)	30	102.6	2.41	22 (73.3)	7 (23.3)	1 (3.3)	0 (0.0)
T5: 5.5 years	202	102.2	1.72	167 (82.7)	34 (16.8)	1 (0.5)	0 (0.0)	21	103.7	2.97	13 (61.9)	6 (28.6)	1 (4.8)	1 (4.8)	25	103.0	2.11	17 (68.0)	7 (28.0)	1 (4.0)	0 (0.0)
CPI ➔				Normal	Mild	Moderate	Severe				Normal	Mild	Moderate	Severe				Normal	Mild	Moderate	Severe
Age at assessment	*N*	Mean	SD	*n* (%)	*n* (%)	*n* (%)	*n* (%)	*N*	Mean	SD	*n* (%)	*n* (%)	*n* (%)	*n* (%)	*N*	Mean	SD	*n* (%)	*n* (%)	*n* (%)	*n* (%)
T0: birth	315	78.9	3.54	315 (100.0)	0 (0.0)	0 (0.0)	0 (0.0)	33	78.2	4.54	33 (100.0)	0 (0.0)	0 (0.0)	0 (0.0)	32	79.2	2.97	32 (100.0)	0 (0.0)	0 (0.0)	0 (0.0)
T1: 7 weeks	315	79.3	4.41	312 (99.0)	3 (1.0)	0 (0.0)	0 (0.0)	33	81.0	5.04	31 (93.9)	2 (6.1)	0 (0.0)	0 (0.0)	32	82.5	5.33	29 (90.6)	3 (9.4)	0 (0.0)	0 (0.0)
T2: 6 months	295	82.2	5.37	272 (92.2)	16 (5.4)	5 (1.7)	2 (0.7)	33	83.8	6.10	28 (84.8)	4 (12.1)	1 (3.0)	0 (0.0)	32	85.3	5.94	27 (84.4)	3 (9.4)	1 (3.1)	1 (3.1)
T3: 12 months	281	79.7	4.46	274 (97.5)	7 (2.5)	0 (0.0)	0 (0.0)	31	81.5	4.94	30 (96.8)	1 (3.2)	0 (0.0)	0 (0.0)	31	82.6	4.76	29 (93.5)	1 (3.2)	1 (3.2)	0 (0.0)
T4: 24 months	253	78.4	4.13	250 (98.8)	3 (1.2)	0 (0.0)	0 (0.0)	29	81.3	4.67	28 (96.6)	1 (3.4)	0 (0.0)	0 (0.0)	30	81.0	4.30	28 (93.3)	2 (6.7)	0 (0.0)	0 (0.0)
T5: 5.5 years	202	78.8	3.82	202 (100.0)	0 (0.0)	0 (0.0)	0 (0.0)	21	80.5	4.41	21 (100.0)	0 (0.0)	0 (0.0)	0 (0.0)	25	81.3	4.42	23 (92.0)	2 (8.0)	0 (0.0)	0 (0.0)

ODDI (%): normal <104, mild 104–107, moderate 108–111, severe ≥112; CPI (%): normal <90, mild 90–94, moderate 95–99, severe ≥100

#### Course of DP

The predictive model for ODDI showed a significant interaction for the parameter time point (*P* < 0.0001), positional preference (*P* < 0.0001), and their interaction (*P* < 0.0001), but not for ODDI at birth (*P* = 0.55). Therefore, the ODDI at birth did not influence the value of ODDI at later time points. The group without positional preference showed an almost stable ODDI over time. Both groups with positional preference showed a strong increase of ODDI at 7 weeks of age and then a gradual decrease over time. However, in children allocated to PPT, the decrease was earlier than in children without intervention, as shown by a significant interaction effect at 6 and 12 months of age. The differences between groups 2 and 3 are small. The outcome of the linear mixed model analysis of the prospective ODDI (DP) is demonstrated in Fig. 2 and Table 3.

**Table 3:** Estimated group differences for ODDI at 7 weeks, 6 months, 24 months and 5.5 years

Estimated difference	Group differences (with 95% CI)	*P* value
Mean ODDI group 3 - mean ODDI group 1		
7 weeks of age	2.58 (1.88; 3.27)	<0.0001
6 months of age	2.69 (1.99; 3.38)	<0.0001
12 months of age	2.01 (1.31; 2.72)	<0.0001
24 months of age	0.57 (−0.15; 1.28)	0.12
5.5 years	0.51 (−0.26; 1.28)	0.19
Mean ODDI group 3 - mean ODDI group 2		
7 weeks of age	−0.27 (−1.19; 0.65)	0.56
6 months of age	1.38 (0.45; 2.30)	0.004
12 months of age	0.64 (−0.30; 1.58)	0.18
24 months of age	−0.45 (−1.41; 0.51)	0.36
5.5 years	−1.10 (−2.16; −0.04)	0.04
Mean ODDI group 2 - mean ODDI group 1		
7 weeks of age	2.85 (2.17; 3.54)	<0.0001
6 months of age	1.32 (0.63; 2.00)	0.0002
12 months of age	1.37 (0.67; 2.07)	0.0001
24 months of age	1.02 (0.30; 1.74)	0.006
5.5 years	1.61 (0.79; 2.42)	0.0001

#### Course of DB

The predictive model for CPI showed a significant interaction for the parameter time point (*P* < 0.0001), positional preference (*P* < 0.001), and CPI at birth (*P* < 0.0001), but not for the interaction term between time and positional preference (*P* = 0.13). CPI at later time points was strongly influenced by the CPI at birth. There was a change in CPI over time, but the pattern was the same for all groups: an increase in CPI at 6 months, followed by a slow decrease to values comparable to the initial values at birth. The group without positional preference had lower scores at all of the time points. Overall the differences between groups are small (Fig. 3 and Table 4).

**Table 4:** Estimated group differences for CPI at 7 weeks, 6 months, 24 months, and 5.5 years

Estimated difference	Group differences (with 95% CI)	*P*-value
Mean CPI group 3 - mean CPI group 1		
7 weeks of age	3.24 (1.59; 4.89)	0.0001
6 months of age	2.94 (1.29; 4.59)	0.0005
12 months of age	2.62 (0.96; 4.28)	0.002
24 months of age	1.88 (0.21; 3.55)	0.027
5.5 years	1.64 (−0.09; 3.38)	0.063
Mean CPI group 3 - mean CPI group 2		
7 weeks of age	1.21 (−0.99; 3.42)	0.28
6 months of age	1.16 (−1.04; 3.37)	0.30
12 months of age	0.96 (−1.26; 3.18)	0.39
24 months of age	−0.42 (−2.67; 1.82)	0.71
5.5 years	0.77 (−1.58; 3.12)	0.52
Mean CPI group 2 - mean CPI group 1		
7 weeks of age	2.02 (0.40; 3.66)	0.015
6 months of age	1.78 (0.15; 3.41)	0.032
12 months of age	1.66 (0.01; 3.30)	0.049
24 months of age	2.30 (0.63; 3.97)	0.007
5.5 years	0.87 (−0.91; 2.65)	0.34

## Discussion

This is the first prospective study of the course of skull shape in healthy newborns with a 5.5-year follow-up. The course of skull deformation is favourable; at 5.5 years of age, CPI is within the normal range for all children, whereas the course of plagiocephaly differs: 80% of the ODDI scores are within the normal range, 19% in the mild range, and only 1% in the moderate to severe range. About 20% of the children scored outside the normal range, which seems to be clinically relevant.

Children with positional preference and DP allocated to PPT showed a rapid decrease of DP, measured at 6 and 12 months, but did not decrease further at 2 and 5.5 years. Remarkably, the children with positional preference and DP allocated to the no PPT group showed a similar result for DP measured at 2 and 5.5 years of age. Overall, the mean DB at 5.5 years of age more or less reached the values of the initial means of DB at birth.

The strengths of the study are the prospective design starting with a healthy population of newborns, the embedded randomised controlled trial regarding the effects of PPT and the use of a reliable and valid primary outcome measurement (plagiocephalometry (PCM)) [38, 42–45]. Also, the use of the (multi-level) linear mixed model analyses of the follow-up data provides a realistic view on the course of skull development and deformation.

This study has several limitations. The definition of muscular torticollis differs in the international literature, which might be confusing in interpreting and comparing studies on asymmetry in infancy. Kaplan et al. [23] categorised congenital muscular torticollis (CMT) into three types: (1) postural CMT presents as the infant’s postural preference but without muscle or passive ROM restrictions and is the mildest form; (2) muscular CMT presents with sternocleidomastoid muscle tightness and passive ROM limitations; and (3) SCM mass CMT, the most severe form, presents with a fibrotic thickening of the sternocleidomastoid muscle and passive ROM limitations. In The Netherlands, the entity of congenital muscular torticollis concerns Kaplan CMT types 2 and 3, and not type 1, which concerns the ordinary postural torticollis [23, 39–45]. In our study, we included type 1 and excluded types 2 and 3.

In the initial study, PPT was performed between 2 and 6 months of age in the intervention group. Having had PPT probably plays a minor role in the development of the skull in the following 5 years. The effect of PPT on skull shape appears to disappear at 24 months of age; therefore, it might have been better to have used PCM outcome to randomise to intervention. When we developed and constructed the initial study, we used the hypothetical rationale that positional preference always occurs before skull deformation. Also, of course, skull deformation remains longer, even when positional preference has disappeared.

From the initial cohort of 380 children, 152 children were lost to follow-up. The general characteristics and PCM measurement values of the children who left the study at each of the time points before T5 were compared with the children who remained in the study. We analysed and compared the skull measurement characteristics at every time point before the last measurement, when the (later) lost to follow up children were still in the longitudinal cohort and were measured. Therefore, we expect that the children lost to follow up did not bias the outcomes, but this can be discussed, because there can be other reasons why parents did not meet the invitation for the final measurement. The dropout percentages differ between groups, but the differences between group 2 (PPT) and group 3 (no PPT) are very small and acceptable.

A limitation discussion point is the use of the cut-off points of ODDI ≥104% for DP and of CPI ≥90% for DB. Some authors discussed the use of cut-off points in skull deformation and it is obvious that the use of other cut-offs will provide other prevalences of severity [19, 20]. The cut-off points were based on a statement in the plagiocephalometry reliability study [41] and were similar to the plagiocephalometry cut-off points used in another recent intervention study regarding skull deformations (HEADS helmet study) [44, 45]. Also, the dichotomy of DP and DB can be discussed. Meyer-Marcotty et al. suggested using a continuum rather than differentiating between the presence or absence of skull deformation, because of the overlapping criteria of DP and DB [28].

Although the course of skull deformation in newborns seems to be favourable, not all of the children with DP (ODDI ≥104%) at 24 months of age fully recovered at 5.5 years of age. At 5.5 years of age, ODDI ≥104% was established in 17.3% (35/202) of the no positional preference group and in 34.8% (16/46) of the positional preference groups.

We have to realise that the conclusions have to be considered cautious and could not be generalised, especially not for children in other countries. In The Netherlands, the efforts to reduce positional preference and skull deformation became more and more structural in the last decennia, so it could have influenced the small differences in outcome.

Positional preference at 7 weeks of age seems to be an important determinant of DP in clinical decision making influencing tailored treatment. Therefore, it is necessary to coach parents in handling and stimulating their child, especially in the early months of life and to focus on children with positional preference, which corresponds to the conclusions of the studies by Aarnivala et al. [1] and Cavalier et al. [10]. A short period of PPT is effective in the earlier reduction of DP [9, 43], especially when it is started before 3 months of age [44]. This may reduce parental fear and increase self-efficacy. None of the included children got helmet therapy. We did not suggest helmet therapy. Van Wijk et al. discussed the effect of helmet treatment as a conclusion of the HEADS study [45], which had also the practical implication that advises to suggest helmet therapy decreased further.

Hutchison et al. found in their follow-up study of DP cases (measured with the HeadsUp method and almost similar cut-off points) that 39% did not revert to normal range at 3–4 years of age [20]. Their findings regarding the recovering of DB were comparable with our study findings [20]. Roby et al. found a prevalence of DP in teenagers in a cross-sectional study of 1.1% and a prevalence of DB of 1.0% [33], which may suggest further recovery in the following years. This is not in line with our findings on the outcome of DP at 5.5 years and is maybe due to the difference in measuring methods; anthropometric calliper measurements are difficult to compare with PCM and HeadsUp measurements, but this has to be considered as a speculation. Furthermore, differences in the prevalence of skull deformation are probably based on the chosen cut-off points or on the use of different measuring methods for skull deformations.

Future studies have to focus on moderate to severe cases of deformational plagiocephaly (ODDI ≥108% at 7 weeks of age), to advise the parents properly in different stages of skull asymmetry (tailored care). Children with severe and/or progressive skull deformations, which do not recover or stay stable in a typical predictable pattern, deserve special attention and alertness. By starting tailored parent counselling and PPT for a short period in children with persistent positional preference early, most of the initial skull deformations may be avoided [10, 43, 44]. Differential diagnostics are indicated to rule out craniosynostosis, especially in progressive skull deformation and facial asymmetry.

## Conclusions

The course of skull deformation (DP and DB) in newborns is favourable in most children in The Netherlands, especially concerning DB. The deformation recovers to acceptable values in nearly all children at 5.5 years of age. Medical resource consumption may be reduced by providing early tailored parent counselling taking into account natural recovery. One should be alert in those cases where the recovery is not progressing as expected.

## Electronic supplementary material

ESM 1 (DOCX 16 kb)

## Abbreviations

CPI: Cranio proportional index
DB: Deformational brachycephaly
DP: Deformational plagiocephaly
ODDI: Oblique diameter difference index
PCM: Plagiocephalometry
PPT: Paediatric physical therapy intervention
