# Supplementary material for: The course of skull deformation from birth to 5 years of age: a prospective cohort study
Source: Eur J Pediatr. 2016 Nov 4;176(1):11–21. doi: 10.1007/s00431-016-2800-0 (PMC5219011; doi:10.1007/s00431-016-2800-0)
Supplement: Supplementary file 1 — (DOCX 16 kb) [file 431_2016_2800_MOESM1_ESM.docx]

### Addendum to “The Course of Skull Deformation from Birth to 5-Years of Age; A Prospective Cohort Study”

### Standardized paediatric physical therapy intervention program and usual care program

The program for the experimental group consisted of exercises to reduce positional preference and to stimulate motor development. The intervention consisted of counselling of parents on (counter-) positioning, handling and nursing of their child, supported by a leaflet, with basic preventive pieces of advice. Parents received extensive education concerning the possible positional preference sustaining factors and practical pieces of advice. Preventive and curative guidelines with accompanying educational material have been designed and used in the counselling of parents on positioning, handling and nursing of the infant. These guidelines did not contradict with the recommendations on the sleeping position of infants in the Dutch equivalent of the “Back to sleep” campaign. The parents of the control group received only the leaflet with basic preventive advices, without further education or instructions to intervene (usual care program). They were told that there was no evidence for better outcome with treatment and they could contact an independent paediatric physical therapist, when they were worried about the development of a persistent positional preference, DP and/or motor development.

The program consisted of a maximum of eight sessions of paediatric physical therapy. In the first month, these sessions were weekly. In the second and third months, every two or three weeks. The second and fifth sessions were always at the child’s home address. All sessions were planned between 7 weeks and 6 months of age. Paediatric physical therapy was stopped when positional preference did no longer occur during day and night, when awake and asleep, when the parents showed to have incorporated all advices and exercises in daily handling, and when there were no indications for motor developmental problems (delay or asymmetries).

Every therapy session of the program was prescribed in detail. The paediatric physical therapy program consisted of mainly positioning, handling and facilitating activities or exercises, all opposite to the directions of the observed positional preference. Tummy time was crucial in this program. Antigravity movements in prone positions when awake were stimulated based on time and frequency (earlier, more frequent and longer playing in prone position). The essential characteristic of the program to prevent or diminish positional preference and therewith decrease the prevalence or severity of deformational plagiocephaly, is the incorporation of all pieces of advice into all daily activities as playing, nursing, changing and dressing, feeding and sleeping and the motivation and compliance of the parents. First, full active cervical range of motion is pursued. Second, symmetrical motor development is stimulated in order to let the child turn his head spontaneously to both side and not having pressure on the same spot of the occipital skull. Playing in prone and adequate side laying positions is very important. Tummy time is advised to do as early, as long and as frequent as possible, strictly under supervision. Antagonists of the muscles, which are responsible in sustaining positional preference, are specially trained.
